# Evaluation of splicing efficiency in lymphoblastoid cell lines from patients with splicing-factor retinitis pigmentosa

**Published:** 2008-12-18

**Authors:** Lenka Ivings, Katherine V. Towns, M.A. Matin, Charles Taylor, Frederique Ponchel, Richard J. Grainger, Rajkumar S. Ramesar, David A. Mackey, Chris F. Inglehearn

**Affiliations:** 1 Section of Ophthalmology and Neuroscience, Leeds Institute of Molecular Medicine, University of Leeds, St James’s University Hospital, Leeds, United Kingdom; 2 Department of Statistics, University of Leeds, Leeds, United Kingdom; 3 Section of Musculoskeletal Disease, Leeds Institute of Molecular Medicine, University of Leeds, St James’s University Hospital, Leeds, United Kingdom; 4 Wellcome Trust Centre for Cell Biology, University of Edinburgh, Edinburgh, United Kingdom; 5 Department of Human Genetics, University of Cape Town Medical School, Cape Town, South Africa; 6 CERA, University of Melbourne, Royal Victorian Eye and Ear Hospital, Melbourne, Victoria, Australia

## Abstract

**Purpose:**

Retinitis pigmentosa (RP) is caused by mutations in a variety of genes, most of which have known functions in the retina. However, one of the most perplexing findings of recent retinal genetics research was the discovery of mutations causing dominant RP in four ubiquitously expressed splicing factors. The aim of this study was to use lymphoblast cell lines derived from RP patients to determine whether mutations in two of these splicing factors, PRPF8 and PRPF31, cause measurable deficiencies in pre-mRNA splicing.

**Methods:**

cDNA was prepared from lymphoblastoid cell lines derived from RP patients bearing mutations in the splicing factor genes and controls, grown under a variety of conditions. Introns representing the U2 and U12 intron classes, with both canonical and noncanonical donor and acceptor sequences, were analyzed by real-time PCR to measure the ratio of spliced versus unspliced transcripts for these introns. In addition, plasmids encoding the retinal outer segment membrane protein-1 (*ROM-1;* exon 1 to exon 2) gene, both in the wild-type form and with mutations introduced into the splice donor sites, were transfected into cell lines. The spliced versus unspliced cDNA ratios were measured by real-time RT–PCR.

**Results:**

Splicing of four canonical U2 introns in the actin beta (*ACTB)*, glyceraldehyde-3-phosphate dehydrogenase (*GAPDH)*, *PRPF8,* and retinitis pigmentosa GTPase regulator (*RPGR)* genes was unaffected in PRPF8 mutant cells. However, the splicing efficiency of *RPGR* intron 9 was significantly decreased in PRPF31 mutant cell lines. In contrast, a consistent decrease in the splicing efficiency of all U12 and noncanonical U2 introns was seen in PRPF8, but not in PRPF31, mutant cells, with statistical significance for *STK11* intron 3.

**Conclusions:**

In spite of the ubiquitous expression patterns of the genes implicated in splicing factor RP, no pathology has yet been documented outside the retina. The observed differences in splicing efficiency described herein favor the hypothesis that these mutations may have a subpathological effect outside the retina. These observations argue against a defect in some yet to be discovered additional function of these proteins and support the alternative hypothesis that this form of RP does indeed result from aberrant splicing of retinal transcripts.

## Introduction

Retinitis pigmentosa (RP) is the most common form of hereditary retinal degeneration, occurring in around 1 in 3,500 people [1]. It is characterized by progressive degeneration of the peripheral retina (leading to night blindness), loss of the peripheral visual fields, and an abnormal electroretinogram. RP is clinically and genetically heterogeneous, with all three modes of Mendelian inheritance: autosomal dominant RP (adRP), autosomal recessive RP (arRP), X-linked RP (xlRP), as well as mitochondrial inheritance running in families. To date some 47 loci have been identified for nonsyndromic RP and for these 36 of the causative genes have been identified (see RetNet for full list). These include genes encoding components of the phototransduction cascade and the visual cycle, by which the chromophore component of rhodopsin is recycled, as well as retinal transcription factors, structural proteins, and proteins thought to be involved in intracellular transport within photoreceptors [2]. In addition, mutations in four ubiquitously expressed splicing factors, pre-mRNA processing factor 8 (PRPF8 [3]), PRPF31 [4], PRPF3 [5], and PIM1 associated protein (PAP-1) [6,7] have also been described in dominant RP patients [8]. Patients with splicing factor mutations do not appear to exhibit any disease phenotype other than retinal degeneration, and the basis of this specificity remains to be determined.

Splicing is a complex process that results in the precise excision of introns from pre-mRNA by a macromolecular structure called the spliceosome [9,10]. The majority of introns are spliced by the major (U2-dependent) spliceosome, which consists of auxiliary protein factors and four small nuclear ribonucleoprotein particles (snRNPs): U1, U2, U5, and U4/U6. However, a small percentage of introns (about 1 in 700 for primates) [11] are spliced by the minor (U12-dependent) spliceosome, which differs from the major spliceosome in four of the snRNPs it contains (U11, U12, U4atac, and U6atac with canonical U5). Signals in intron and exon sequences further define intron and exon recognition and the execution of splicing. In both U2 and U12 introns, canonical (GT/AG) and various noncanonical consensus splice site sequences are used. U12-dependent introns are characterized by a more highly conserved branch site and lack of a polypyrimidine tract [12,13]. It has been suggested that the splicing of U12-dependent introns may be a rate-limiting step in gene expression [14].

PRPF8 is a 220 kDa protein that is highly conserved both in sequence and size, varying between 220 and 280 kDa in different organisms. It is a component of the U5snRNP and U5·U4/U6 tri-snRNP [15]. PRPF8 can also be photochemically cross-linked to the 5′ splice site, the branchpoint and the 3′ splice site in the pre-mRNA [16–18] and to the U5 and U6 snRNAs [19,20]. PRPF8 is therefore thought to be at the catalytic center of the spliceosome [21,22]. This close association with crucial RNA sequences and proteins in the spliceosome suggests that PRPF8 could directly affect the function of the catalytic core, perhaps acting as a splicing cofactor. Unlike most other known splicing factors, as a component of the U5 snRNPs, PRPF8 participates in both U2 and U12 splicing [15].

Mutations in PRPF8 cause a severe form of dominant RP [3]. To date, 16 different mutations, including missense, premature stop and deletions, have been identified [3,23–26]. They cluster in a highly conserved region within the last exon, encoding the C-terminus of the protein, making it unlikely that these transcripts will be removed by nonsense-mediated decay [27]. This is supported by real-time PCR analysis that has shown similar expression of both wild-type and mutant alleles in cell lines from RP patients carrying a nonsense mutation [28]. This remarkable clustering of mutations and the relatively high number of missense changes could imply that the mutations alter a specific domain of particular importance to retinal function. Crystal structural analysis of the last 273 amino acids of *Caenorhabditis elegans* PRPF8 shows that the RP mutations are contained in a C-terminal extension that is not thought to affect the overall structure of this domain [29]. However, it is hypothesized that the C-terminal peptide (2310–2335) constitutes a direct binding surface between PRPF8 and other partners, and mutations on this surface could therefore lead to disruption of critical protein–protein interactions necessary for function [29], a finding recently confirmed by Boon et al. [30].

PRPF31 is a 61 kDa splicing factor first identified in yeast [31]. It is a U4/U6 snRNP-associated protein that promotes tri-snRNP association between U4/U6 snRNP and U5 snRNP by direct interactions with a 102 kDa U5-specific protein [32]. At present, there are 19 known RP-causing mutations in PRPF31. Only three of these are missense changes while the remainder encode severely truncated or grossly abnormal transcripts (six deletions ranging from one base pair to the whole gene, five splice-site mutations, two insertion/deletion events, one duplication, and one insertion) [24,33–41]; these changes may be targeted by nonsense-mediated decay [27]. In support of this, allele-specific measurement of PRPF31 transcripts have revealed a strong reduction in the expression of mutant alleles, with no truncated proteins being detected. Blocking of nonsense-mediated mRNA decay restored the amount of mutant PRPF31 mRNA but did not restore the synthesis of mutant proteins [42]. This suggests that mutations in PRPF31 cause RP due to haploinsufficiency, an observation further supported by the finding that high-expressing alleles of *PRPF31* from the normal parent compensate for a potentially RP-causing mutation on the opposite chromosome [43]. This phenomenon explains the unusually high level of nonpenetrance associated with RP due to mutations in *PRPF31* [44].

Three hypotheses can be put forward to explain how mutations in splicing factors might cause RP in the absence of any other defect. The first is that mutations specifically affect the splicing of one or several retina-specific genes. An obvious target would be rhodopsin (RHO), which makes up 80% of the protein content of the rod outer segment [45]. The constant process of disc shedding and renewal of the rod outer segments means that demand for *RHO* mRNA might be high, which could be met through new synthesis as well as a function of mRNA stability. Consistent with this first hypothesis reduced splicing of an exon 3–4 minigene construct of rhodopsin was associated with cells transfected with a mutated version of PRPF31 [46].

A second hypothesis would be that mutations cause a generalized splicing deficiency but this is only pathological in the fast-metabolizing, end-differentiated retina [47]. This could be consistent with a defect in the translocation of these splicing factor proteins to the nucleus, as proposed by Deery et al. [48], or with a deficit in U12-dependent spliceosome function, as proposed by McKie et al. [3].

A third hypothesis is that PRPF8 and PRPF31 have another function unrelated to mRNA splicing, as proposed for another disease-implicated splicing factor, survival of motor neuron 1 (SMN1) [49]. The finding of mutations in four spliceosomal components leading to adRP would however tend to suggest involvement of the whole tri-snRNP, rather than separate components of it, in this alternative function.

Hypotheses one and two, that the RP-causing mutations cause splicing deficiency, may be more consistent with the haploinsufficiency mechanism proposed for PRPF31 mutations, rather than the dominant negative effect implied by the clustered missense mutations found in PRPF8. In this study we compared the efficiency with which U2 and U12 introns (both canonical and noncanonical) were spliced in cell lines derived from RP patients carrying PRPF8 or PRPF31 mutations and controls. The study also aimed to determine whether RP-causing mutations resulted in measurable splicing defects in lymphoblastoid cell lines derived from these patients.

## Methods

### Cell culture

Samples of blood from individuals carrying mutations in PRPF8 were collected by D. Mackey (Melbourne, Australia) and R. Ramesar (Cape Town, South Africa; see Table 1 for details) and deposited with European Collection of Cell Cultures (ECACC; Salisbury, UK), where cell lines were then prepared by EBV transformation. All individuals with PRPF8 mutations had RP. Individuals recruited were otherwise healthy. Peripheral venous blood was obtained from patients and other family members by venepuncture using lithium heparin or sodium citrate vacutainers. Samples were stored and transported at room temperature for a maximum of 5 days before immortalisation with EBV. All blood samples were collected from patients after obtaining informed consent from each participant and receiving local ethics committee approval. The remaining cell lines were obtained directly from ECACC. These included cell lines from patients and asymptomatic carriers with PRPF31 mutations and also unaffected relatives of PRPF31 mutation carriers, who were used as controls. The individuals from whom these lines were prepared were again otherwise healthy. Cells were grown in RPMI1640 medium with L-glutamine supplemented with 10% heat-inactivated FBS (Gibco, Carlsbad, CA) at 37 °C in 5% CO_2_ and maintained according to ECACC protocols.

**Table 1:** Cell lines used in this study with their corresponding mutations and patient characteristics

**Group**	**Cell line**	**Mutation**	**Sex**	**Age**
Control	AG0318	Control	M	22
	AG0300	Control	F	32
	AG0309	Control	F	34
	AG0267	Control	M	46
	AG0326	Control	F	58
	AG0261	Control	F	77
	AGO296	Control	F	56
PRPF31 Carrier	AG0353	Exon 11, 11 bp Del, nt 1115–1125	M	22
	AG0311	Exon 11, 11 bp Del, nt 1115–1125	F	23
	AG0298	Exon 11, 11 bp Del, nt 1115–1125	M	58
PRPF31 Severe	AG0306	Exon 11, 11 bp Del, nt 1115–1125	F	47
	AG0325	Exon 11, 11 bp Del, nt 1115–1125	M	56
	HG0003	IVS 6 +3 A>G	F	62
	HG0005	Whole gene deletion	F	65
	AG0316	Exon 11, 11 bp Del, nt 1115–1125	F	72
PRPF8	HG0007	H2309P	F	33
	HG0008	H2309P	F	67
	HG0009 HG0011 HG0012	H2309P	F	72
		H2309R	F	60
		H2309R	F	30

Samples were collected by D.Mackey (Melbourne, Australia), or R.Ramesar (Cape Town, South Africa), or obtained from the ECACC as stated in Materials and Methods.

### RNA extraction and cDNA synthesis

Total RNA was extracted from pelleted cells using the Qiagen RNeasy extraction kit according to the manufacturer’s protocol. The process included a 15-min treatment with DNaseI (Qiagen) to digest any contaminating genomic DNA. Next, 1 µl of RNA was mixed with 1 µg random primer (Invitrogen, Glasgow, UK) and 10 µl nuclease-free water. This mixture was incubated at 70 °C for 10 min and chilled on ice. To each reaction was added 8 µl of master mix: 4 µl 5X Moloney Murine Leukemia Virus Reverse Transcriptase (M-MLV RT) buffer, 2 µl 0.1 M dithiothreitol (DTT), 2 µl 10 mM deoxynucleosides (dNTPs; all Invitrogen), and 0.25 µl RNAsin (Promega, Hampshire, UK). This then was equilibrated at 37 °C for 2 min. Next, 0.5µl Moloney Murine Leukemia Virus (M-MLV) reverse transcriptase (Invitrogen) was added to each reaction, followed by incubation at 37 °C for 1 h and at 95 °C for 5 min. For each cell line a control cDNA synthesis without reverse transcriptase was performed and the presence of genomic DNA was excluded by failure to PCR with GAPDH primers shown in Table 2.

**Table 2:** Primers used in real time PCR analysis

**Primer name**	**Sequence (5′-3′)**	**Primer name**	**Sequence (5′-3′)**
GAPDH ref F	AACAGCGAGACCCACTCCTC	APRT ex 2–3 F	GGGCCGCATCGACTACAT
GAPDH ref R	CATACCAGGAAATGAGCTTGACAA	APRT ex 2–3 R	AGCCCAGTCCAAGCTCCTG
18S F	CGGCTTTGGTGACTCTAGATAACC	APRT in 2–ex 3 F	AACCAGGTACCCCTTGCCAC
18S R	AAGTTGATAGGGCAGACGTTCG	APRT in 2–ex 3 R	CAAAGAGGAAGCCTCGGGAG
β-actin ex 2–3 F	CCTGGCACCCAGCACAA	PLCδ ex 3–4 F	AGATCATCCACCACTCAGGCTC
β-actin ex 2–3 FR	CCGATCCACACGGAGTACTT	PLCδ ex 3–4 R	TGTCAGCTTTTCGCAAGCAG
β-actin in 2-ex 3 F	AGCTGTCACATCCAGGGTCC	PLCδ in 3–ex 4 F	CTTGGTAGGTTCCAGGGTTCCT
β-actin in 2-ex 3 FR	CCGATCCACACGGAGTACTT	PLCδ in 3–ex 4 R	TTGTCAGCTTTTCGCAAGCA
PRPF8 ex 25–26 F	AGGCAAGAGGCCATTGCTC	STK11 ex 3–4 F	TGAGGAGGTTACGGCACAAAA
PRPF8 ex 25–26 R	GATTCGAGGAATGCCACGAT	STK11 ex 3–4 R	GCCACACACGCAGTACTCCAT
PRPF8 ex 25–in 25 F	AAGAGCCCATTGCTCAGAACA	STK11 in 3–ex 4 F	AGCTGTGTGTCCTTAGCGCC
PRPF8 ex 25–in 25 R	ACTCCACACGGTTCAAAGGC	STK11 in 3–ex 4 R	GCACACTGGGAAACGCTTCT
RPGR ex 10–11 F	TCTATCAGCACGTATGCGGC	C3G ex 15–16 F	TCCCCAGAGGAGCTCATCAA
RPGR ex 10–11 R	AAACAAGCAGAAAGGCCAAGAG	C3G ex 15–16 R	TTGCTGACGCGCTTCTTG
RPGR in 9–ex 10 F	TTCTGTGGATTTATGCTGCAGG	C3G in 15–ex 16 F	GCCGACCACATGGCTATTTC
RPGR in 9–ex 10 R	TTTTGCCACACCACGATGAG	C3G in 15–ex 16 R	GACGCGCTTCTTGAATGTGTC
ZNF198 ex 8–9 F	GCGCCAAGTGATATTCAGTTGA	AP2A1 ex 19–20 F	CCTCGGTGCAGTTCCAGAAT
ZNF198 ex 8–9 R	TGCTGCAGAACTGATGCACTTT	AP2A1 ex 19–20 R	GCTTGGTCTGCACAGCCAG
ZNF198 in 8–ex 9 F	TGCTTCTGTAAAAGTGGCGTGT	AP2A1 ex 19–in 19 F	CTCGGTGCAGTTCCAGAATTTC
ZNF198 in 8–ex 9 R	TTTGCTGCAGAACTGATGCAC	AP2A1 ex 19–in 19 R	GACGTGGAGAGGCAGGAGG

The table lists the primer name and corresponding amplified exon. Primers were designed using Primer Express 1.5 (ABI) as described in Materials and Methods.

### Real-time PCR

Ten introns (in ten different genes) were selected for analysis to represent both U2 and U12 introns with canonical and noncanonical donor and acceptor sites. Primer Express 1.5 software (ABI Prism 7000 User’s Guide, Applied Biosystems, Foster City, CA) was employed to design primer pairs. Pairs spanned two adjacent exons or an exon and adjacent intron and were used to amplify a product of 80–140 bp, with an annealing temperature between 58 and 60 °C (see Table 2 for complete primer list). Primer pair concentrations were optimized for real-time PCR analysis according to (ABI, Applied Biosystems) recommendations. GAPDH and 18S were used as endogenous references. cDNAs of highly expressing transcripts were diluted to obtain comparable levels of cycle threshold (C_t_). Real-time PCR was performed using the SYBR Green PCR Core Reagents Kit (ABI, Applied Biosystems) as described by Ponchel et al. [50], and all samples were run in duplicate on an ABI PrismTM 7700 Sequence Detector System. Expression levels of each exon-exon and exon-intron were normalized to GAPDH using a standard ΔC_t_ method.

### Statistical analyses

The ratio of spliced to unspliced RNA was calculated for each data point as relative amount of exon-exon product divided by relative amount of exon-intron product. Descriptive statistics on the ratios for each gene and cell line were performed and graphs were produced in Excel. For each cell line the replicates were replaced by their respective means for four groups: normal cells; those from PRPF31 mutation carriers without RP; those from affected patients with PRPF31 mutations; and those with PRPF8-RP. These were compared using one-way ANOVA to detect any overall difference between the four groups, and the LSD post-hoc multiple comparison test was applied to examine individual intergroup differences, using the SPSS statistics base 17.0, SPSS inc, Chicago, IL. In addition, multivariate tests were performed in SPSS to analyze inherent group differences between the control and the mutant groups for U2 and U12 introns, and further, a one-sided bootstrap test was performed.

### Serum starvation

Cells were incubated in a serum-free medium for 24 h. The medium was then replaced with serum-containing medium. Samples were taken at 0, 2, 4, 6, 12, 24, and 72 h after another addition of serum. Total RNA was then extracted and cDNA synthesized for real-time PCR analysis as described in the previous section.

### Transfections

A plasmid minigene containing mouse retinal outer segment membrane protein-1 (Rom1) intron 2 was constructed by Dr J. Wu (Saint Louis University, St. Louis, MO) and was obtained as a gift from Professor Eric Pierce, Scheie Eye Institute, Philadelphia, PA. Two donor site sequence changes were introduced using the Stratagene (La Jolla, CA) site-directed mutagenesis kit (according to manufacturer’s instructions as described above), which altered the wild-type canonical GT site to AT and GA. Cells were transfected using the Effectene transfection reagent (Qiagen) following the manufacturer’s protocol.

## Results

### Pre-mRNA splicing in lymphoblastoid cell lines derived from patients with PRPF8 and PRPF31 adRP

To determine whether mutations in splicing factors associated with RP cause detectable splicing defects in non-retinal tissues, we obtained lymphoblastoid cell lines from four groups of individuals: RP patients carrying the H2309P or H2309R mutation in PRPF8; those with the R372_A375delfs mutation in PRPF31 who have severe RP; those carrying the PRPF31 R372_A375delfs PRPF31 mutation but not manifesting RP [4]; and controls (see Table 1).

Ten exon-intron-exon sequences were chosen for analysis. These included four “house-keeping” genes containing U2 introns with consensus splice sites, β-actin (*ACTB*), glyceraldehyde 3-phosphate dehydrogenase (*GAPDH*), the splicing factor *PRPF8* gene itself, and *RPGR*, mutations in which also lead to RP [51–53]. Also included were two genes containing U2 introns with non-consensus splice sites, *ZNF198* (a zinc-finger protein encoding gene), and adenine phosphoribosylase (*APRT*) . Finally four genes were selected containing U12 introns, one with a consensus GT/AG splice site from phospholipase C delta (*PLCδ*), and three with non-consensus splice sites, serine-threonine kinase 11 (*STK11*), ras guanine nucleotide releasing factor C3G and adaptor-related protein complex 2 α-1 (*AP2A1*; see Table 3). The U12 introns were chosen from a database of confirmed U12 introns [54].

**Table 3:** Genes selected for analysis of splicing

**Intron type**	**Splice site sequence**	**Gene**	**Exons**
U2	GT/AG	β-actin	2–3
	GT/AG	GAPDH	7–8
	GT/AG	PRPF8	25–26
	GT/AG	RPGR	10–11;in 9–ex 10
	GC/AG	ZNF198	8–9
	GC/AG	APRT	2–3
U12	GT/AG	PLCδ	3–4
	AT/AC	STK11	3–4
	AT/AC	C3G	15–16
	AT/AG	AP2A1	19–20

This table summarizes gene intron type, splice site sequence and amplified exon for each gene used in analysis.

**Figure 1:**
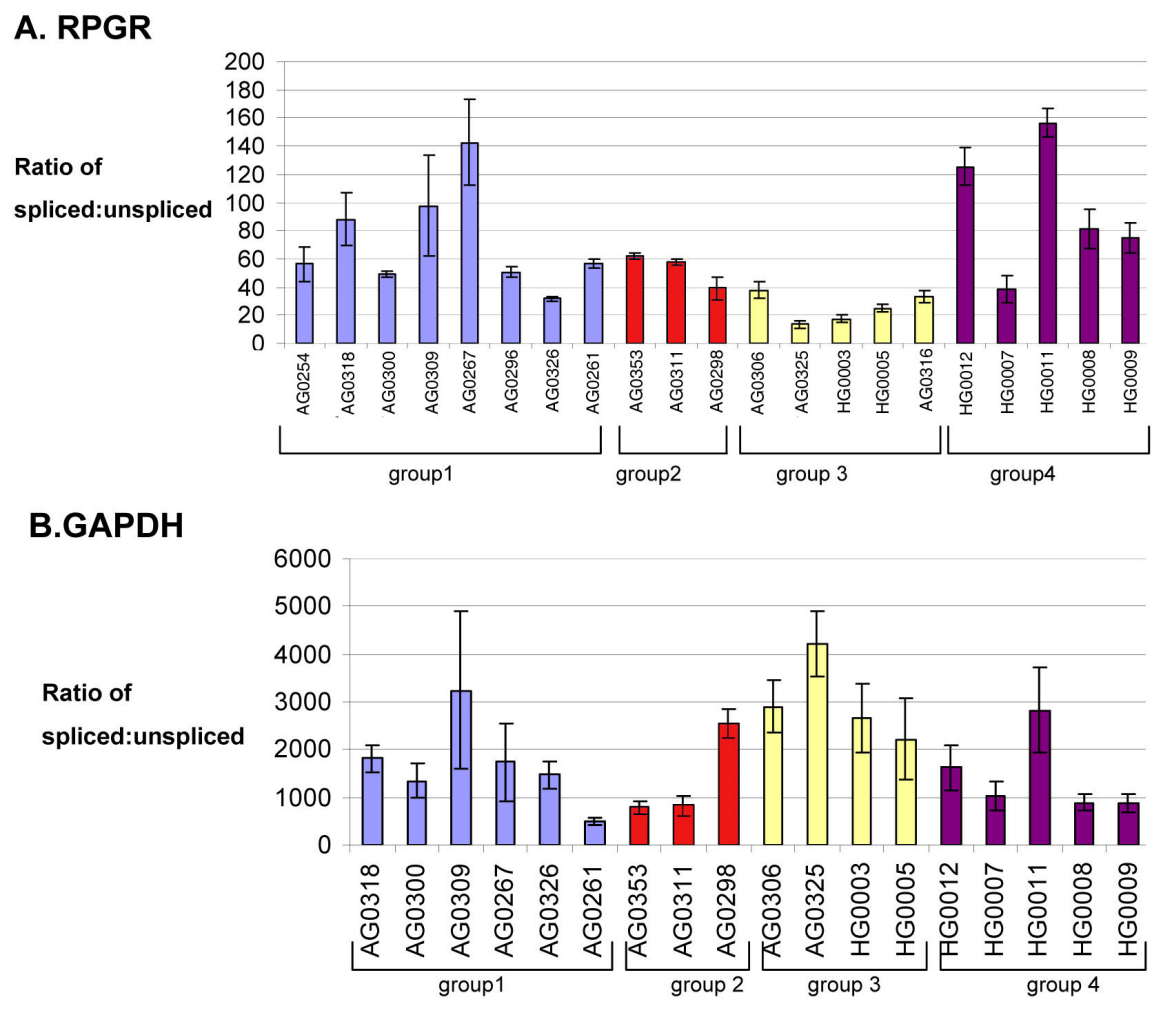
**Splicing ratios of U2.** Splicing ratios of U2 containing introns *RPGR* (**A**) and *GAPDH* (**B**) RNA in cell lines derived from control and splicing-factor mutated cell lines. Group 1 represents controls, group 2 represents PRPF31 carriers, group 3 represents PRPF31 severe, and group 4 represents PRPF8 cell lines. The splicing ratios were obtained as described in the Methods section. Means from four to six repeat experiments are shown ±SEM. For individual cell line characteristics see Table 1.

**Figure 2:**
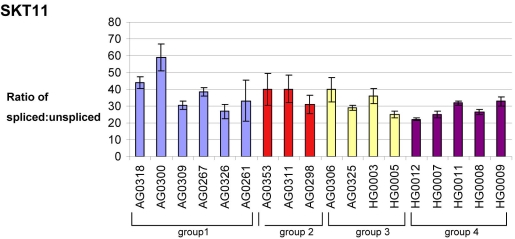
**Splicing ratio of U12.** Splicing ratio of U12 containing intron *STK11* RNA in cell lines were derived from control and splicing-factor mutated cell lines. Group 1 represents controls, group 2 represents PRPF31 carriers, group 3 represents PRPF31 severe, and group 4 represents PRPF8 cell lines. The splicing ratios were obtained as described in the Methods section. Means from four to six repeat experiments are shown ±SEM. For individual cell line characteristics see Table 1.

The splicing efficiency of these introns in control and patient cell lines was determined by examining ratios of spliced versus unspliced transcripts using real-time PCR. Relative levels of exon-exon product were divided by relative levels of exon-intron product to produce an estimate of splicing efficiency. Values for the three groups of cell lines were then compared with values obtained in control cell lines, and results analyzed using ANOVA and LSD tests as described in the previous section. Results are shown in Figure 1, Figure 2, and Table 4.

**Table 4:** Ratios of spliced to unspliced RNA in groups of control and splicing factor mutant cell lines for selected pre-mRNA introns

**Intron and splice site**	**Gene**	**Control**	**PRPF31 carrier**	**PRPF31 severe**	**PRPF8**
U2 GT/AG	ACTIN	328	406	315	402
U2 GT/AG	GAPDH	1683	1385	**2993 p<0.05**	1433
U2 GT/AG	PRPF8	198	173	184	140
U2 GT/AG	RPGR	78	105	**23 p<0.05**	95
U2 GC/AG	ZNF198	154	166	128	124 ↓
U2 GC/AG	APRT	8	10	7	6.5 ↓
U12 GT/AG	PLCd	18	19	21	9 ↓
U12 AT/AC	SKT11	39	37	32	**28 p<0.05 ↓**
U12 AT/AC	C3G	17	24	9	13 ↓
U12 AT/AG	AP2A1	36	39	40	29 ↓

Significant differences obtained by the LSD test are highlighted in bold. A significant increase in splicing efficiency was observed for GAPDH in PRPF31 severe cell lines. In contrast a significant decrease in splicing efficiency was observed for RPGR splicing in the same cell line. In PRPF8 cell lines a significant decrease in splicing efficiency was seen for SKT11, as well as an overall decline in splicing for all U12 introns and non-canonical U2 introns.

### U2 introns

No significant differences in splicing ratios were observed between control and PRPF8 mutant cell lines for any U2 introns. There were also no significant differences in splicing ratios of U2 introns between control cell lines and those deriving from asymptomatic patients with PRPF31 mutations. In contrast, PRPF31 mutant cell lines from patients with a severe phenotype showed significantly lower splicing ratios for *RPGR* with respect to controls (Figure 1) and significantly higher splicing ratios for *GAPDH*. These data are summarized in Table 4.

### U12 introns

None of the splicing ratios assayed for U12 introns in PRPF31 mutant cell lines, either those from RP patients or from asymptomatic carriers, showed significant differences from those obtained in control cell lines. However, PRPF8 mutant cells showed significantly decreased splicing ratios for intron 3 of SKT11 with respect to controls (Figure 2). *AP2A1*, *PLC-delta*, and *C3G* introns did not show statistically significant differences between control and PRPF8 mutant cell lines; however, a general trend could be seen toward a decrease of splicing ratios in PRPF8 mutants compared to controls for all U12 introns. This trend also extended to the two noncanonical U2 introns. These data are summarized in Table 4.

### Multivariate tests

Multivariate tests were performed to investigate these findings. These tests failed to ascertain the apparent decrease in splicing ratios in PRPF8 mutant cells for U12 introns. This could be due to the sample sizes being too small, or to the parametric test relying on incorrect assumptions. Therefore a bootstrap test was performed, in which the data were resampled, a test statistic was computed, and the results were compared to the data test statistic. The one-sided p-value for the U12 introns comparing controls versus PRPF8 mutants was 0.067, whereas the p-value for controls versus PRPF31 severe was much higher (0.30). This confirmed that the trend toward decreased splicing deficiency seen for PRPF8 mutant cell lines (which just fell short of significance) was not reproduced in PRPF31 mutant cell lines.

### Analysis of splicing ratios under high metabolic demand

One possible explanation for the retina-specific manifestations of splicing-factor RP is the high metabolic rate of photoreceptors [47] and the consequent inability of the mutated PRPF8 or PRPF31 to cope with the accompanying splicing demands; this might become especially apparent for rate-limiting U12 intron splicing [14]. To investigate whether splicing is affected during periods of high demand on the splicing apparatus, we starved control and patients’ cell lines of serum for 24 h. The splicing efficiency was investigated by real-time PCR following the reintroduction of serum (at 0, 2, 4, 6, 12, 24, and 72 h), which would be expected to result in a substantial increase in the rate of cell division and growth. No significant differences were found (data not shown).

### Study of the splicing of transfected retina-specific gene, *Rom-1*

In a further attempt to determine if splicing could be affected by conditions of high splicing demand, we transfected cell lines with plasmids encoding ROM-1 (exon 1 to exon 2), both in the wild-type form and with mutations introducing a noncanonical splice donor sites (canonical GT mutated to noncanonical AT and GA). The spliced versus unspliced ratios of all constructs were again measured by real-time RT–PCR. No significant differences in splicing efficiency between the control and splicing factor mutant cell lines for any of these constructs were detected (data not shown).

## Discussion

These experiments aimed to distinguish between two hypotheses: that splicing factor mutations cause RP due to a retina-specific defect, either in splicing or in some other function involving these proteins; or that splicing factor RP results from a defect in splicing that is subpathological elsewhere in the body but which leads to a cumulative defect in the metabolically active, irreplaceable retinal tissue. To distinguish between these hypotheses, we assayed the ratio of spliced to unspliced RNA as a measure of splicing efficiency in lymphoblastoid cell lines derived from splicing factor RP patients and normal control cell lines. This assay was applied to a series of introns representing a range of intron types.

Results did reveal significant differences in splicing ratios in these cells, but these differences were not consistent between the different splicing factor genes. In cells from PRPF31-RP patients, we observed a decrease in the splicing efficiency of *RPGR* intron 9 and an increase in the splicing efficiency of *GAPDH* intron 7. These are both U2 introns with canonical donor and acceptor sites. In contrast, cells from patients with PRPF8-RP showed no significant differences in splicing ratios for U2 introns but showed a consistent downward trend in splicing efficiency of all U12 and noncanonical U2 introns assayed (statistical significance was only attained for intron 3 of *STK11*). Cells from asymptomatic carriers of PRPF31 mutations revealed no significant differences in splicing ratio compared with control cells, consistent with the findings of Vithana et al. [43], who showed that high expression of the second allele of PRPF31 compensated for the haploinsufficiency. The lack of consistency between findings in the cell lines from patients with the two different mutant genes for splicing factor may be the result of the small number of cell lines assayed, together with the relatively high level of individual variation in splicing efficiency between cell lines.

The finding of a difference in splicing efficiency in an intron of *RPGR* may be significant. RPGR is a putative guanine nucleotide exchange factor that has been localized to the Golgi region [55], and is thought to be important for maintaining the polarized protein distribution across the connecting cilium of the photoreceptors [56]. Mutations in *RPGR* (*RP3*, Xp21.1) are responsible for up to 70% of X-linked retinitis pigmentosa [51] and also cause X-linked cone-rod dystrophy (*CORDX1*) [52] and atrophic macular degeneration [53]. However, RPGR is ubiquitously expressed, and several alternatively spliced isoforms of RPGR have been found in the eye and other tissues, resulting in product sizes ranging between 5 and 20 kb [57,58]. One of these isoforms contains a retina-specific exon, ORF15, which has been found to be a mutational hotspot in RP patients [59,60]. The intron tested herein is present in this and other isoforms expressed in the eye, but is absent from others also present in the eye. Correct splicing of *RPGR* may be particularly important in the retina; mutations in PRPF31 may have a negative effect on the splicing of *RPGR* pre-mRNA, which could potentially contribute to the RP phenotype in these patients. In the light of this finding it may now be informative to look at splicing efficiency in other ubiquitously expressed genes which are mutated in RP.

The one significant difference in splicing between control and PRPF8 mutant cells was found within the U12 intron-containing gene *STK11*. Furthermore, although no other significant differences were observed, there was a consistent trend toward an overall decrease in splicing efficiency of all U12 introns tested. U12 splicing is thought to be the rate-limiting step in the processing of any transcript containing a U12 intron [14]. In the fast-metabolizing retina, with a corresponding high demand placed on the splicing machinery, a reduction in splicing efficiency of U12 intron-containing transcripts could become significant over time, while other tissues remain unaffected. Microarray analysis by Gamundi et al. [28] in PRPF8 mutant lymphoblastoid cell lines showed downregulation of one U-12 type gene, *SLC12A7*, but failed to ascertain a pattern of downregulation of the minor U12-dependent splicing genes as a group. However that study used microarray analysis to examine transcript levels, whereas this study examined intron specific splicing efficiency, potentially a more sensitive method for detecting such an effect.

It is also interesting to note that the splicing ratio of *GAPDH* intron 7 was significantly increased in cells from PRPF31-RP patients relative to controls. GAPDH, as well as being a major enzyme of the glycolytic pathway, has several other functions, such as regulation of gene expression [61]. More important, GAPDH has been found to have a role in the apoptosis of retinal cells [62]. The endpoint in many forms of retinal degeneration is apoptosis of the retinal photoreceptors, so this difference could also contribute to the development of an RP phenotype.

Given the relatively small number of cell lines investigated and the small number of splice sites chosen, the substantial variation in splicing efficiency between cell lines (control and disease groups) and the inconsistency of differences found between the different genes for RP, these data must be interpreted with caution. Nevertheless, this study has recorded statistically significant differences in splicing efficiency in cell lines from patients with two different mutated genes for splicing-factor RP. This favors the hypothesis that RP may indeed result from a global deficit in splicing, detectable outside the retina, but which only causes pathology in the retina. Further elucidation of the mechanisms underlying splicing-factor RP will play an important role in the search for therapeutic agents.
